# Impact of Mindfulness Training on Physiological Measures of Stress and Objective Measures of Attention Control in a Military Helicopter Unit

**DOI:** 10.1080/10508414.2015.1162639

**Published:** 2016-05-10

**Authors:** Anders Meland, Kazuma Ishimatsu, Anne Marte Pensgaard, Anthony Wagstaff, Vivianne Fonne, Anne Helene Garde, Anette Harris

**Affiliations:** ^a^ Norwegian Armed Forces Medical Services, Institute of Aviation Medicine, Oslo, Norway; ^b^ Graduate School of Health Care Sciences, Jikei Institute, Osaka, Japan; ^c^ Norwegian School of Sport Sciences, Oslo, Norway; ^d^ National Research Centre for the Working Environment, Denmark, and Department of Public Health, University of Copenhagen, Copenhagen, Denmark; ^e^ Faculty of Psychology, University of Bergen, Bergen, Norway

## Abstract

**Objective**: This study sought to determine if mindfulness training (MT) has a measurable impact on stress and attentional control as measured by objective physiological and psychological means.

**Background**: Periods of persistent, intensive work demands are known to compromise recovery and attentional capacity. The effects of 4-month MT on salivary cortisol and performance on 2 computer-based cognitive tasks were tested on a military helicopter unit exposed to a prolonged period of high workload.

**Methods**: MT participants were compared to a wait list control group on levels of saliva cortisol and performance on a go–no go test and a test of stimulus-driven attentional capture. Participants also reported mental demands on the go–no go test, time of wakeup, sleep duration, quality of sleep, outcome expectancies, physical activity level, self-perceived mindfulness, and symptoms of depression and anxiety.

**Results**: The results from a mixed between–within analysis revealed that the MT participants compared to the control group had a larger pre to post increase in high- and low-cortisol slopes, and decrease in perceived mental demand imposed by the go–no go test.

**Conclusion**: MT alleviates some of the physiological stress response and the subjective mental demands of challenging tasks in a military helicopter unit during a period of high workload.

Human beings have a capacity to adapt to and endure prolonged periods of high workloads. However, repeated, excessive, or prolonged energy mobilization without sufficient rest might contribute to physical disease, increased mortality, reduced well-being, and deficits in executive functioning in normal populations (Cohen, Janicki-Deverts, & Miller, 2007; Liston, McEwen, & Casey, 2009; McEwen, 1998) and in military cohorts (Jha et al., 2015; Jha, Stanley, Kiyonaga, Wong, & Gelfand, 2010; Schnurr, Lunney, Bovin, & Marx, 2009; Vasterling et al., 2006). Therefore, there is a growing concern about the long-term effects of physical and psychological demands combined with prolonged high workloads associated with military deployments. A report by the Rand Corporation states that deployments are longer and more frequent than in conflicts such as the Vietnam and Persian Gulf wars and that there are shorter intervals between deployments (Tanielian & Jaycox, 2008). Under these circumstances, one should continually strive to minimize the burden on the personnel, knowing that there will still be times when little can be done to reduce workload involved in mission completion.

It is far from clear which strategies work best to conserve energy and maximize recovery when reduction in external workload is not an option. Mindfulness training (MT) is a psychological intervention that is suggested to improve situational awareness (Bishop et al., 2004), reduce systemic arousal, and increase parasympathetic activity without compromising alertness (Ditto, Eclache, & Goldman, 2006; Tang, Holzel, & Posner, 2015; Tang et al., 2009). A capacity to remain both calm and alert is not a combination humans have naturally inherited through the millennia. Therefore, MT has been suggested to be of particular value for several groups striving for excellence in taxing environments (Fornette et al., 2012; Jha et al., 2010; Sappington & Longshore, 2015).

## MINDFULNESS TRAINING AND THE STRESS RESPONSE

Neurological research suggests that the stress-reducing effects of MT result from adaptation, consolidation, and attribution processes (Hôlzel et al., 2011). People often react automatically to challenges and find ways to distract or suppress unwanted experiences. These mental strategies are strenuous, they can increase parasympathetic nervous system activity, and they can make us more vulnerable to environmental stressors (Gross, 2002). By contrast, MT encourages one to adopt a state of restful alertness to present-moment experience, stressful or not, making no effort to control thoughts or feelings that might emerge (Bishop et al., 2004).

This shift in strategy could be stress-reducing and less demanding in itself, because by default it involves a more present-centered awareness (Brewer et al., 2011). Over time this strategy is thought to improve people’s ability to tolerate and cope with negative emotional states, by extinguishing activation responses and avoidance behaviors previously elicited by these stimuli (Baer, 2003). The process could be further stimulated by a change in the way one perceives a situation, which is known to be of importance to adjust or habituate to stressors (Lazarus & Folkman, 1984). Another possibility is that the automaticity of physiological activation caused by a stressor is short-circuited when an individual believes he or she can cope with the stressor, a so-called positive response outcome expectancy (PROE; Ursin & Eriksen, 2004).

## CORTISOL AS A BIOLOGICAL MARKER OF RECOVERY

Cortisol, the end product of the hypothalamic–pituitary–adrenal (HPA) axis, is one of the biological mediators providing energy to cope with daily demands, and is regarded as a reliable marker of the physiological stress response (Pruessner et al., 1997). The cortisol hormone triggers the release of glucose in the body and is an important part of energy mobilization and expenditure, but also the wear and tear of the body. Cortisol levels are subject to short-term fluctuations caused by acute stressors, and a slower 24-hr cortisol cycle, closely related to metabolic rate. Cortisol levels are highest during the second half of the night, with a peak level in the morning after awakening, and decline throughout the day with the nadir around midnight (McEwen, 1998; Wilhelm, Born, Kudielka, Schlotz, & Wûst, 2007). Acute exposure to a taxing environment would normally lead to higher secretion of cortisol in healthy individuals, but repeated energy mobilization without sufficient recovery depletes bodily resources and could lead to exhaustion and lower cortisol secretion. Prolonged periods of high demands have therefore been associated with lower morning cortisol levels, a blunted cortisol awakening response (CAR), and higher evening cortisol values (Kristenson et al., 2012; O’Leary, O’Neill, & Dockray, 2015; Schlotz, Hellhammer, Schulz, & Stone, 2004). Thus, the CAR and cortisol slopes from morning to evening might all be valuable indicators of the capacity to respond to or recover from stressful stimulation. CAR is a measure of the responsiveness to wake-up of awakening; it is calculated as the difference between cortisol levels at wake-up and 30 min after wake-up. High slope is a measure of the maximal dynamic, because it is calculated as the difference between the peak cortisol values 30 min after wake-up to bedtime. Low slope is calculated from the wake-up values to bedtime and therefore excludes the extreme value related to the wake-up response (Kristenson et al., 2012). Moreover, higher CAR scores and steeper slopes could indicate normal metabolism and sufficient recovery after a stressful stimulus, whereas lower CAR and blunted slopes could indicate a lack of recovery.

## THE STRESS-BUFFERING EFFECTS OF MT

Stress-buffering effects of MT have been found in highly stressed community workers (Nyklìcêk, Mommersteeg, Van Beugen, Ramakers, & Van Boxtel, 2013) and nurses (Klatt, Steinberg, & Duchemin, 2015), and patient populations exposed to prolonged stressors (e.g., patients with HIV, psoriasis, pain, or chronic inflammation; Cohen et al., 2007). In a study by our group, we also found that 12 months of MT improved self-reported mindfulness, attentional control, and arousal regulation in a high-performance combat aircraft unit (Meland, Fonne, Wagstaff, & Pensgaard, 2015). These studies are consistent with the proposed theory that MT has potential restorative effects on individuals exposed to prolonged periods of high demands. There is a lack of controlled studies testing the effects of MT using objective measures of stress and attentional control, especially during prolonged periods of high workloads.

The aim of this study was therefore to test the restorative effect of MT in a healthy high-performance cohort using objective measures of the stress response, recovery, and attentional control. We capitalized on the rare opportunity afforded by access to personnel at two military helicopter units during a prolonged period of high workload. One group received 4 months of MT, and the other served as a wait list control group. We expected that the MT would effectively increase self-perceived mindfulness. Due to the cumulative fatigue induced by the personnel’s prolonged exposure to high workload at pretest, we hypothesized that the restorative effects of MT would produce an increase in CAR and higher wake-up values while evening values remained low, thereby producing steeper cortisol slopes. Based on the proposed change in attentional strategy, we expected that the MT group would commit fewer errors on the go–no go test and increased sensitivity to the target on attentional capture test. We also expected that the MT group would find the go–no go test less demanding to perform after the intervention, compared to the control group. As the sample was comprised exclusively of selected high-performing individuals we did not expect to find significant changes in already low levels of anxiety, worry, or depression.

## METHOD

### Experimental Design

A pretest and posttest with nonrandom assignment were used to investigate the effects of MT on stress reduction and attentional control. The choice of intervention group was based on the geographical availability of MT instructors delivering the intervention. Data from questionnaires, saliva samples, and scores on computerized tests were collected before and after the intervention period.

### Variables

Group affiliation was the single independent variable in this experiment, and there were a number of dependent variables, including levels of saliva cortisol, measures of performance on a go–no go test, and a test of stimulus-driven attentional capture test. We also measured self-reported mindfulness, mental demand on the go–no go test, anxiety, depression, and daily positive response outcome expectancies. Length and quality of sleep, amount of physical activity, and demographics were also documented.

#### Cortisol

Three measures of cortisol were derived from the data. The CAR was calculated by subtracting the 30 min postwaking value from the waking value. The high and low slopes were calculated by subtracting the bedtime value from the waking value and 30 min postwaking values, respectively (Kristenson et al., 2012)

#### Sustained Attention to Respond Task

The Sustained Attention to Respond Task (SART; Robertson, Manly, Andrade, Baddeley, & Yiend, 1997) is sensitive to transitory reductions in attention and the response inhibition element of executive function, yet minimizes demands on other cognitive processes such as memory, planning, and general intellectual effort. Two scores were derived from the SART: number of errors of commission (responses to the rare no-go digit, 3) and reaction times (RTs) to frequent go stimuli.

#### Attentional Capture Task

To reduce the burden on participants we designed a shortened version of the Attentional Capture Task (ACT; Theeuwes & Chen, 2005) based on a previous experiment (not published). The ACT measures the vulnerability for automatic and reflexive attentional capture. The attentional capture is created by a task-irrelevant peripheral stimulus (i.e., a red blink) that flashes for 60 msec equally often at one of the six positions prior to the target appearance. The attentional capture effect is measured in five conditions according to the presence and position of the distractor: (a) no distractor: no red-blink; (b) cued condition: red blink on target position; (c) Distance 1: red blink closest to target; (d) Distance 2: red blink second closest to target; and (e) Distance 3: farthest away from the target. The task took 15 min to complete. Two scores were derived from the ACT on all five conditions: mean RTs and sensitivity (*d*’) in which the vertical target was discriminated from the horizontal target. Sensitivity was calculated from “hits” and “false alarms” (Stanislaw & Todorov, 1999).

#### Mindfulness

The Norwegian version of the Five Facet Mindfulness Questionnaire (FFMQ; Dundas, Vøllestad, Binder, & Sivertsen, 2013) consists of 39 items rated on a five-point Likert scale ranging from 1 (*never true*) to 5 (*always true*). The subdimensions of the FFMQ had reasonable internal consistency: observation (α = .78), description, (α = .84, after removing Item 7), acting with awareness (α = .87), nonjudgmentality (α = .87), and nonreaction (α = .61, after removing Item 21 due to low reliability).

#### Mental Demand on the SART

The SART is a demanding task where error rates are commonly high (Robertson et al., 1997). The subjective demand performing the SART was measured using a subscale of the NASA Task Load Index (NASA–TLX). The participants rated one item on a 20-point Likert scale ranging from 1 (*not demanding at all*) to 20 (*very demanding*; Hart & Staveland, 1988).

#### Anxiety

The Norwegian Sports Anxiety Scale (SAS–n; Abrahamsen, Roberts, & Pensgaard, 2006) is a multidimensional, sport-performance, trait-anxiety inventory that is often used to investigate high-performance groups. Responses are given on a four-point scale ranging from 0 (*not at all*) to 3 (*very much*). The three subdimensions had reasonable internal consistency: somatic anxiety (α = .81), worry (α = .88), and concentration disruption (α = .75).

#### Depression

We used a Norwegian version of the one-dimensional 10-item Hopkins Symptom Checklist (Strand, Dalgard, Tambs, & Rognerud, 2003). Responses are given on a four-point scale ranging from 0 (*not at all*) to 3 (*very much*) and had reasonable internal consistency (α = .89).

#### Daily Positive Response Outcome Expectancies

To measure PROE, participants were asked to respond to the following question: “How well do you expect to cope with the challenges of today?” The question was answered on the mornings when saliva samples were taken and responses were given on a 10-point scale ranging from 1 (*not very well*) to 10 (*very well*).

#### Sleep

On the days of saliva sampling, participants logged their number of hours of sleep and their quality of sleep on a five-point scale ranging from 1 (*very good*) to 5 (*very bad*).

#### Physical Activity

Participants gave an estimate of how often they worked out per week pre- and postintervention on a scale of from 1 (*more than 2–3 passes*), 2 (*2–3 passes*), 3 (*1–2 passes*), 4 (*less than 1 pass*), and 5 (*hardly ever*).

#### Demographics

Age, military experience, and whether respondents were living with a partner or not were recorded.

### Participants

The sample consisted of all available personnel at two military helicopter units (*N* = 40) during their preparations for redeployment to an aeromedical mission in a conflict area. The squadrons were comparably organized, performed similar tasks, and had similar working hours, responsibilities, and characteristics (see Table 1).

**TABLE 1:** Characteristics of the Mindfulness Training (MT) Group and Control Group

	*MT Group (*n* = 25)*	*Control Group (*n *= 15)*
Mean age (*SD*)	35 years (13), range = 18–62	40 years (10), range = 26–56
Mean military experience (*SD*)	15 years (13), range = 2–42	20 years (11), range = 7–35
Married or living with partner	16	13
Number of aircrew	16	13
Number of technicians/administrators	9	2

At the time of the experiment the personnel had been, and still were, exposed to considerable workload as a result of deployment to the conflict area combined with intensive pre- and postdeployment work. Unpublished reports from Air Force headquarters indicated that personnel in both squadrons were showing clear signs of attrition and fatigue. The mission had lasted several years when the study started, and at posttest it was still unclear how much longer the mission would last.

### Equipment

Saliva was collected using the Salivette sampling device with cotton roll. The stimuli for the cognitive tests were presented on a laptop with a 15–in. liquid crystal display (DellTM LatitudeTM E6520) located approximately 60 cm from participants. A laptop running SuperLab 4.5 software (Cedrus Corporation) controlled the timing of the events, generated stimuli, and recorded response times.

### The Intervention

The intervention followed the guidelines for the Mindfulness Stress Reduction Program (MBSR; Kabat-Zinn, 1994). It started with a 10-hr comprehensive introductory course, which was followed by weekly 3-hr sessions and twice-weekly, 20-min audio-guided personal MT sessions. To increase MT practice time, participants were encouraged to carry out everyday activities that they usually did on autopilot more mindfully (e.g., working out, talking, listening, eating, walking, driving, etc.). All MT instructors had a minimum of 10 years of meditative practice and were formally accredited MT instructors at the Scandinavian Centre for Awareness training (www.scat.no). The intervention was carefully designed to fit a high-performance environment and participants’ partners were also offered MT (for further details on the MT, see Meland et al., 2015).

### Procedure

Participants who were scheduled for the experiment received a short briefing about the experiment accompanied by an information letter. The MT was part of the daily planned activity at the intervention squadron and was therefore mandatory. Participants provided written informed consent to participation in the research project. The stated purpose of the experiment was “to gain a better understanding of the physiological and psychological effects of MT in high-performance cohorts.” Workers who declined participation at recruitment (*n* = 3) were excused from data collection procedures but otherwise followed the same program as the study participants, giving a response rate of 95%. The study was approved by the Norwegian Committee for Medical Research Ethics.

Data collection was performed at the squadrons’ home base, and every effort was made to collect the data at the same time of day and week for both squadrons. To correct for seasonal differences in daylight due to the geographical locations of the squadrons, the data were collected in February and May for the MT group and in April and August for the control group.

#### Cortisol Sampling

Participants collected saliva samples over 2 days on waking, 30 min after waking, and at bedtime. Participants were instructed to wake according to their regular schedule and collect the first sample in bed (instruction: “as soon as you open your eyes”), the second sample later (instruction: “after 30 min, but before eating, drinking, or brushing teeth”), and the last one at bedtime (instruction: “the time at which you try to go to sleep, prior to brushing your teeth”). Salivettes were refrigerated for a maximum of 2 days and returned to the investigators where they were frozen (minimum −18°C) until analysis. The same procedures were used at pre- and postmeasurements.

Analysis of hormones in saliva was done using liquid chromatography tandem mass spectrometry (LC–MS/MS; Jensen, Hansen, Abrahamsson, & Nørgaard, 2011). The detection limit was 0.26 nmol/l. To test equivalence between analysis, reference samples at two levels (3.22 nmol/l and 9.03 nmol/l) were analyzed with every 14 samples. Westgard control charts (Westgard, Barry, Hunt, & Groth, 1981) were used to document that the method remained under statistical and analytical control.

In accordance with current guidelines (Stalder et al., 2016), all samples taken more than 5 min outside the time window in the protocol were excluded (*n* = 4), and delayed wake-up samples (> 20 min postwaking) were turned into 30-min postwaking samples (*n* = 5). The data were checked for outliers, but no unlikely cortisol levels were detected. Cortisol levels were averaged across the 2 days for each of the three scheduled collection times on a per-individual basis. To maximize the volume of data available for analysis, all participants with at least one valid pre- and postintervention saliva sample for a given time point were included in the analysis. Logarithmic transformations of cortisol data did not change the results of the analysis, so for ease of interpretation raw data were used in the analysis and presentation of data.

#### Cognitive Tests and Questionnaires

All participants were briefed on the two tests on the morning prior to pretests, and received adaptation and practice, 3 min on the SART and 8 min on the ACT. Questionnaires were filled in at the end of this common session. Participants went back to their regular working positions and met at designated time slots for cognitive testing. To control for daily variations in attention, the time of day for cognitive testing was the same for pre- and posttesting. The cognitive test session started with the SART, followed by the ACT, separated by a 2-min break. A 1-min practice was included in both the SART and ACT, which was not included in the data analysis.

Prior to the SART, participants were instructed to respond to the go stimuli (1, 2, 4, and 9) with a key press and to withhold this response when the no-go stimulus (3) was presented. Prior to the ACT, participants were instructed to discriminate the orientation of the target line (vertical or horizontal) placed inside the diamond, and that the diamond appeared unpredictably but equally likely in one of six positions. Participants were instructed to respond as quickly and accurately as possible in both tests, and that the SART took 5 min to complete and the ACT took 15 min.

### Statistical Analysis

Statistics were calculated using SPSS version 22.0 (IBM Corporation, Armonk, NY). Independent-sample *t*-tests were used to assess baseline group differences. The effects of MT were assessed using mixed-factor analysis of variance (ANOVA), with one between-subject factor (MT group vs. control) and one within-subjects repeated-measures factor (pre- and postintervention). The main effects for time and group and the interaction of these factors (Time × Group) are reported. A significant interaction effect indicates a different development (from pre to post) over time between the groups. The main effect of time reflects changes over time across groups. The main effect of group indicates that the two groups differed when collapsed across the measurement times. Paired-sample *t*-tests were performed to assess the change in mindfulness scores. The significance threshold was set at *p *< .05 for all analyses.

## RESULTS

**FIGURE 1:**
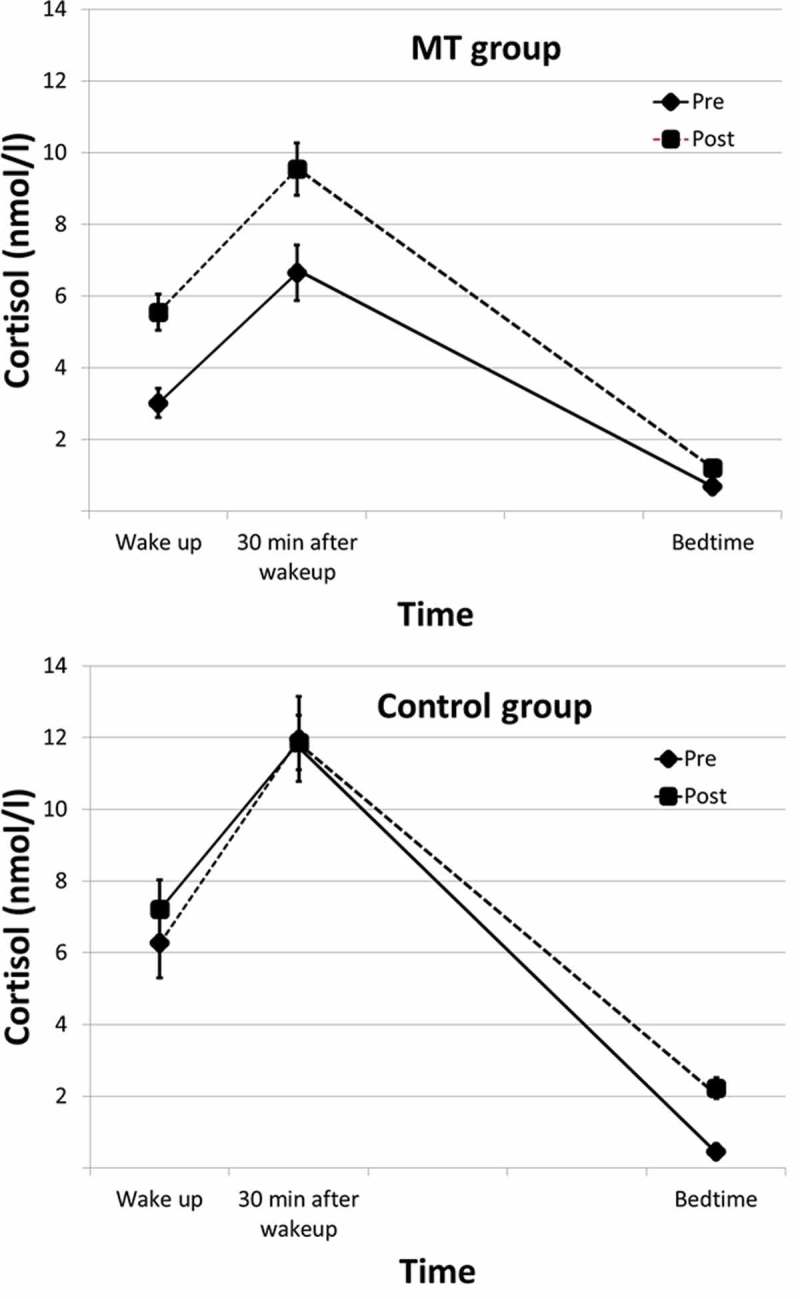
Mean salivary cortisol levels at wake-up, 30 min after wake-up, and bedtime pre- and postintervention in the (a) mindfulness training (MT) group (*n* = 25), and (b) control group (*n* = 15), with error bars showing standard error.

The analysis included 25 participants in the MT group and 15 in the control group. It should be noted that the initial size of the groups was larger. Six MT participants and 2 control group participants did not take any postintervention saliva samples due to change in workplace (*n* = 2), absence due to deployment (*n* = 2), sick leave (*n* = 2), or forgetting (*n* = 2). These were excluded from our analysis, and because there were no baseline differences between participants with and without missing postintervention saliva data, the missing data could be considered random (results not reported). It should also be noted that both high and low slope levels of cortisol and perceived concentration disruption scores were lower in the MT group compared to the control group at baseline (Table 2, Table 3 and Figure 1). No other baseline group differences were found.

**TABLE 2:** Means and Standard Deviations of High and Low Cortisol Slopes, Cortisol Awakening Response, Scores on the Sustained Attention to Respond Test, and Scores on the Attentional Capture Test

	*MT Group (*n* = 25)*				*Control Group (*n* = 15)*					
	Pre		Post		Pre		Post		Time	Time × Group Interaction
	M	SD	M	SD	M	SD	M	SD	F value, *p* value (η^2^)	F value, *p* value (η^2^)
Cortisol										
High slope (nmol/l)	6.0	3.9	8.3	4.1**	10.9	4.1	9.8	2.8**		*F*(1, 38) = 5.537, *p* = .024 (.127)*
Low slope (nmol/l)	2.3	1.9	4.4	2.7**	5.4	3.5	4.6	2.6**		*F*(1, 38) = 6.828, *p *= .013 (.156)*
CAR (nmol/l)	3.4	2.9	4.0	3.1	5.7	4.8	4.6	3.1		
Sustained attention to respond test										
Commission error	19.69	5.21	18.38	5.75	18.07	4.71	17.00	3.23		
RT (msec)	305	66	322	54	326	51	303	38		*F*(1, 38) = 8.202, *p *= .007 (.174)**
Mental demand (rating scale = 0–20)	15.38	3.41	11.25	5.36	16.07	2.27	15.71	2.58	*F*(1, 38) = 10.805, *p *= .002 (.231)**	F(1, 38) = 7.636, *p *= .009 (.175)**
Attentional Capture test										
*d*’ cued condition	1.92	1.06	2.07	1.26	2.58	1.06	2.37	.86		
*d*’ Distance 1	.97	.69	1.40	.97	1.16	.41	1.21	.73	*F*(1, 38) = 4.878, *p *= .033 (.114)*	
*d*’ Distance 2	1.00	.79	1.44	.98	1.27	.44	1.45	.87	*F*(1, 38) = 8.330, *p *= .006 (.180)**	
*d*’ Distance 3	1.16	.94	1.59	1.02	1.34	.83	1.41	.74		
*d*’ No distractor	1.44	.96	1.64	1.08	1.49	.72	1.71	.98		

*Note*. Differences between groups tested with a mixed between–within subjects analyses of variance (Wilks’s Lambda). MT = mindfulness training; CAR = cortisol awakening response; RT = reaction time.

*p < .05. **p < .01.

**TABLE 3:** Means and Standard Deviations of Responses to Sport and Anxiety Scale, Sleep Length, Sleep Quality, Positive Response Outcome Expectancy, Physical Activity, and Wake-Up Time

	*MT Group (*n* = 25)*				*Control Group (*n* = 15)*				
	*Pre*		*Post*		*Pre*		*Post*		
	*M*	*SD*	*M*	*SD*	*M*	*SD*	*M*	*SD*	*Time*
Sport and Anxiety Scale: (rating scale = 0–3)									
Anxiety	.52	.24	.47	.22	.64	.25	.67	.19	
Concentration disruption	.17	.23	.10	.24*	.47	.36	.38	.75*	
Worry	.68	.34	.48	.34	.65	.35	.60	.37	*F*(1, 38) = 5.984, *p *= .020 (.158)*
Depression	.26	.30	.16	.18	.36	.41	.24	.32	*F*(1, 38) = 5.837, *p *= .022 (.154)*
Sleep length (hr)	6.47	.61	6.74	.61	6.71	.83	6.86	1.12	*F*(1, 38) = 5.971, *p *= .020 (.162)*
Sleep quality (rating scale = 1–5)	1.95	.78	1.79	.71	2.50	1.23	2.00	1.04	*F*(1, 38) = 4.910, *p *= .034 (.002)*
Positive response outcome expectancy (rating scale = 1–10)	7.50	1.46	7.60	1.31	7.71	1.07	8.00	.87	
Physical activity (rating scale = 1–5)	2.42	1.12	2.32	0.89	2.29	1.07	2.29	1.20	
Wake-up time (a.m.)	6:21	0:31	6:31	0:34	6:26	0:42	6:33	0:30	

*Note*. Differences between groups tested with a mixed between-within subjects analyses of variance (Wilks’s Lambda). MT = mindfulness training.

*p < .05. **p < .01.

### Mindfulness

Paired-sample *t*-tests revealed a pre to post increase in the observation and description facets of the FFMQ in the MT group, but not in the control group (Table 4).

**TABLE 4:** Pre and Post Means and Standard Deviations of the Different Facets of Mindfulness in Mindfulness Training (MT) Group and Control Group

	*MT Group (*n* = 25)*				*Control Group (*n* = 15)*			
	Pre		Post		Pre		Post	
Facet	M	SD	M	SD	M	SD	M	SD
Observation	3.00	.53	3.21*	.58	2.89	.54	2.74	.45
Description	3.16	.56	3.46**	.57	3.36	.45	3.32	.62
Act with awareness	3.48	.63	3.53	.59	3.19	.59	3.09	.63
Nonjudgmentality	3.79	.65	3.89	.65	3.85	.59	3.76	.66
Nonreactive	3.21	.60	3.36	.43	3.01	.39	2.83	.54

*Note*. Rating scale = 1–5.

*p < .05. **p < .01.

### Cortisol

The mixed-factor ANOVA revealed a Group × Time interaction on two of the cortisol measures (Table 2). The MT group displayed an increase on high and low cortisol slopes, with no change in CAR. This pre–post change is mainly due to increases in morning measures of cortisol in the MT group (Figure 1a), leaving bedtime cortisol levels unchanged. This change in morning levels of cortisol was not found in the control group (Figure 1b).

Significant Group × Time interactions were also found in RT and self-perceived demand on the SART. On the ACT, there were main effects for time in two conditions. Referring to Table 3, there was a main effect for time on the depression scores and the worry subscale of the SAS–n, showing similar pre–post reductions in both groups on these self-perceived measures. There was also a main effect for time on the sleep measures, showing similar increases in sleep length and quality. No other interaction effects were found on any of the other measures.

## DISCUSSION

The aim of this study was to investigate the effects of MT on stress reduction and attentional control in a high-performance cohort during a prolonged period of high-demand work. As hypothesized, we found that the intervention increased two of the five self-perceived facets of mindfulness (i.e., observation and description), confirming that the intervention efficiently affected the degree of mindfulness. We also found steeper cortisol slopes from morning to evening and decreased perceived mental workload on the demanding go–no go test. The findings support the proposal that the stress-reducing effects of MT are related to a more restful, alert, and flexible state of mind, allowing for adaptation and active coping responses (Moore & Malinowski, 2009; Tang et al., 2015). This also extends our previous reports that MT improves self-reported regulation of arousal and attention in already high-performing individuals (Meland et al., 2015).

### Changes Due to MT

There could be several possible explanations to the change in cortisol levels of the MT participants found in this study. Importantly, there were no indications that this change could be attributed to changes in outer workload, positive outcome beliefs, physical activity, time of wake-up, or sleep. We cannot rule out that it is partly related to a statistical artefact, regression to the mean (Bland & Altman, 1994). Still, we believe this is unlikely in this study, because we found pre–post changes in several other measures in the MT group, which were not present in the control group.

The design of the MT intervention could, of course, have played a role in the changes in cortisol, for example the plenary layout of the training sessions. Simply spending more time together in plenary sessions could have led to an increased sense of social support. The stress-reducing effects of social support are documented in demanding occupational settings (Bragard, Dupuis, & Fleet, 2015). It is also possible that spending 3 to 5 hr a week sitting or lying down in itself could have had a relaxing and restorative effect on the participants. However, the fact that we observed changes in self-perceived mindfulness in the MT group suggests that some of the beneficial effects also were due to adoption of specific mindfulness skills.

An attitude of acceptance is known to short-circuit unhelpful thought patterns and rumination (Segal, Wittmann, & Teasdale, 2002; Wells, 2011). However, a pre–post change in the nonjudgmental facet of mindfulness could not be stated. Participants could still have benefited from MT because it increases the extent to which they exposed themselves to the inner turmoil that inevitably accompanies periods of pressure, uncertainty, and high workload. Merely to register stressful thoughts and feelings, no matter attitude, could over time result in adaptation and normalization (Hôlzel et al., 2011). All other things being equal, this could have led to a reduction in the proportion of daily experience perceived as stressful to the MT participants, reducing unnecessary energy mobilization.

Another postulated consequence of MT is that one learns to notice unhelpful automatic thoughts without responding reflexively to them, including those that have previously been regarded as both positive and adaptive. For example, an inner striving to improve is considered an adaptive trait (Stoeber & Otto, 2006), but during periods of prolonged or excess pressure, an automatic tendency to “try harder” or “wishing things were different” could be the source of unnecessary energy expenditure. Successful athletes are known to have confidence and a high drive to excel (Gould & Maynard, 2009). Because the mental skills needed in sports are similar to those in the military (DeWiggins, Hite, & Alston, 2010), this could have been of particular importance to the stress-reducing effects of MT in this study. The stress response might have been further intensified because many of the stressors were beyond the control of the individuals in this study (Lazarus & Folkman, 1984). MT is in fact believed to be especially helpful in circumstances that cannot be controlled (Baer, 2003; Bishop et al., 2004). In sum, instead of striving for control or manipulating and changing thoughts, the MT participants might therefore have learned to identify unhelpful thoughts, and started to respond reflectively to them, rather than reactively. This could have lessened systemic arousal and improved their ability to recover, without a corresponding reduction in outer workload.

As stated, there are several ways MT can lessen the impact of daily stressors and increase recovery in this selected cohort, but common to all of them is that it involves a change in attentional strategy. One way of controlling attention is to force our attention back to a target when our mind wanders, striving to maintain attentional focus and judging oneself when one fails to do so. This attentional strategy has been associated with increased energy expenditure (Gross, 2002), and might over time be exhaustive. In MT one learns to allow attention to rest with alertness on the present-moment experience, gently moving it or bringing it back with no further cognitive processing. This change in attentional strategy is associated with decreased systemic arousal (Tang et al., 2015), and might account for the restorative effects of MT in this study. The lowered perceived demands imposed by the SART found in this study support this explanation and are consistent with Randomized Controlled Trials (RCTs) showing that individuals perform information-processing tasks more efficiently and with less effort after MT (Tang et al., 2015).

The slowing of RTs on the SART in the MT group supports an increased awareness in the MT group, because the opposite (i.e., speeding of RTs on the SART) has been associated with a lack of awareness and habitual responding (Cheyne, Solman, Carriere, & Smilek, 2009). We have previously found that an environmental stressor (i.e., vibration) was associated with shorter RTs on the SART in elite athletes (Ishimatsu, Meland, Hansen, Kåsin, & Wagstaff, 2016), which fits with the speeding up of RTs in the control group. Following this argument, we would have expected a significant decrease in commission errors in the MT group and an increase in the control group. This was not the case in this study.

Becoming more perceptually attuned and sensitive to inner present-moment experience has been suggested to be potentially problematic to some individuals (Segal et al., 2002; Wells, 2011). However, MT did not seem to make the current sample more vulnerable to depression, anxiety, and stimulus-driven distractions, as measured by the ACT.

### Strengths and Limitations

To our knowledge this is the first controlled study of the effects of MT in a high-performing cohort during prolonged high-demand work, using salivary cortisol and performance on tests reflecting attentional capacities important to real-life situations experienced in aviation. The sample size and duration of the intervention should have been sufficient to elicit and detect the putative effects of MT on cortisol secretion (Tang et al., 2015). Although our sample was exclusively male and might therefore be exceptional in some respects, we consider the findings to be reliable and expect they will generalize to other high-performance populations exposed to similar demands.

Although these findings lend further support to the benefits of MT for high-performance individuals, there are a number of limitations to the study. Baseline differences between the groups in the levels of cortisol, not accounted for by differences in the group characteristics, could be regarded as a limitation. This could be due to the natural between-individual difference in cortisol levels (Kudielka, Hellhammer, & Wust, 2009). However, we do not find this limitation too concerning because such differences are accounted for in the within-subjects design. The lack of an active control condition is a more serious limitation, which means we cannot separate the effects caused by MT skills and those attributable to other factors of the intervention. The fact that we also failed to find pre–post improvements in performance on the cognitive tests means that we cannot exclude the possibility that some of the stress-reducing effect of MT is attained through a reduction in overall alertness and situational awareness. Finally, we relied solely on quantitative pre- and postintervention assessments and did not carry out a follow-up assessment or collect verbal reports. This means we lack insight into individual trajectories, cultural differences, and long-term effects of MT. In sum, the findings should be replicated in other high-performance cohorts and if possible include an active controlled condition, longer test duration, follow-up measures, and qualitative interviews.

## CONCLUSION

A 4-month MT program was effective in increasing high and low cortisol slopes, leaving CAR unaffected, and reducing perceived demands on a go–no go test. The program also increased the observation and description aspects of mindfulness, indicating that the restorative effects of MT came through increased exposure to present-moment experience and a more relaxed and flexible mind, less vulnerable to habitual responding.

### Practical Implications

These findings indicate that MT could be implemented specifically to reduce stress in high-workload settings where attentiveness to the task is particularly important. This could be relevant in military and civilian aviation, as well as other high-demand contexts where external workload cannot easily be lessened.

## FUNDING

This research was financially supported by the Norwegian Air Force Flight Safety Inspectorate, Norwegian Armed Forces Medical Service, Institute of Aviation Medicine, and the Norwegian Olympic Training Centre.
